# Italian XEN-Glaucoma Treatment Registry (XEN-GTR): Effectiveness and Safety at 36 Months of XEN45 Implant

**DOI:** 10.3390/jcm13237370

**Published:** 2024-12-03

**Authors:** Francesco Oddone, Gloria Roberti, Sara Giammaria, Chiara Posarelli, Leonardo Mastropasqua, Luca Agnifili, Tommaso Micelli Ferrari, Vincenzo Pace, Matteo Sacchi, Romeo Altafini, Gianluca Scuderi, Andrea Perdicchi, Carmela Carnevale, Antonio Fea, Michele Figus

**Affiliations:** 1 IRCCS—Fondazione Bietti, Via Livenza 6, 00198 Rome, Italy; gloria.roberti@fondazionebietti.it (G.R.); sara.giammaria@fondazionebietti.it (S.G.); carmela.carnevale@fondazionebietti.it (C.C.); 2 Department of Surgical, Medical, Molecular Pathology and of Critical Care Medicine, University of Pisa, Via Savi 10, 56126 Pisa, Italy; chiaraposarelli@gmail.com (C.P.); michele.figus@unipi.it (M.F.); 3 Ophthalmology Clinic, Department of Medicine and Aging Science, University G. D’Annunzio of Chieti-Pescara, Via dei Vestini 29, 66100 Chieti, Italy; mastropa@unich.it (L.M.); l.agnifili@unich.it (L.A.); 4 Regional General Hospital F. Miulli of Acquaviva delle Fonti, Strada Prov. 127, 70021 Acquaviva delle Fonti, Italy; t.micelliferrari@miulli.it (T.M.F.); v.pace@miulli.it (V.P.); 5 Department of Medicine, Surgery and Pharmacy, University of Sassari, Via Roma 151, 07100 Sassari, Italy; matteosacchi.hsg@gmail.com; 6 Ophthalmology Unit, Azienda Ospedaliero-Universitaria di Sassari, Viale San Pietro 43, 07100 Sassari, Italy; 7 Ophthalmology Clinic, Dolo Hospital, Via XIX Aprile, 2, 30031 Dolo, Italy; raltafi@tin.it; 8 Ophthalmology Unit, St. Andrea Hospital, NESMOS Department, University of Rome “Sapienza”, Via di Grottarossa 1035, 00189 Rome, Italy; gianluca.scuderi@uniroma1.it (G.S.); perdicchi@gmail.com (A.P.); 9 Struttura Complessa Oculistica, Città Della Salute e Della Scienza di Torino, Dipartimento di Scienze Chirurgiche-Università Degli Studi di Torino, Via Cherasco 23, 10126 Torino, Italy; antoniomfea@gmail.com

**Keywords:** POAG, XEN45, intraocular pressure, glaucoma surgery, subconjunctival filtration surgery, MIGS

## Abstract

**Objectives**: We evaluated the 3-year effectiveness and safety of XEN45, combined or not with phacoemulsification, in patients from the Italian XEN-Glaucoma Treatment Registry. **Methods**: Data from glaucoma patients who underwent XEN45 alone or combined with phacoemulsification were analyzed. Changes in intraocular pressure (IOP) and the number of ocular hypotensive medications (OHMs) were tested with repeated measures ANOVA in last observation carried forward (LOCF) and per-protocol (PP) analyses. Complete and qualified success (IOP < 18 mmHg and ≥20% IOP reduction from baseline, without or with OHMs) at 36 months and pre- and intraoperative factors predicting surgery failure were explored using survival analysis and Cox proportional-hazard models. Complications rates were evaluated to assess safety. **Results**: The analysis included 239 eyes (239 patients): 144 (60.2%) in the XEN alone and 95 (39.8%) in the XEN+Phaco groups. Overall success was achieved in 164 (68.1%) eyes [113 (68.9%) complete and 51 (31.1%) qualified], without significant differences in success (*p* = 0.07) and survival rates (*p* = 0.46) between groups. At the 36th month, the baseline IOP decreased from a median (IQR) of 23.0 (20.0–26.0) to 15.0 (12.0–17.5) mmHg (*p* < 0.01), with an overall 34.1 ± 20.1% IOP reduction. The mean ± SD number of OHMs decreased from the baseline of 2.7 ± 0.9 to 0.9 ± 1.1 at month 36 (*p* < 0.01). PP and LOCF analyses were comparable. Neither pre- nor intraoperative factors were significantly predictive of surgery failure. In 91 (38.1%) and 57 (23.8%) of the eyes, at least one postoperative complication occurred early (<month 1) and late (≥month 1) during follow-up, respectively, without sequelae. During follow-up, 68 (28.5%) eyes needed at least one needling. **Conclusions:** At 3 years, XEN45, combined or not with phacoemulsification, effectively and safely reduced IOP and OHMs need.

## 1. Introduction

Glaucoma management poses a persistent challenge, requiring a delicate balance between intraocular pressure (IOP) control and preservation of visual function. In addition to the many existing molecules for medical therapy, the introduction of minimally invasive surgical techniques has expanded the options for glaucoma treatment in recent years, with XEN45 Gel Stent (Allergan, an Abbvie company) emerging as a promising option for its ability to provide sustained IOP reduction. Its mechanism of action involves facilitating aqueous humor drainage from the anterior chamber of the eye into the subconjunctival space bypassing the trabecular meshwork, and the less invasive nature of the surgical procedure aims to reduce the risks of vision-threating complications of traditional filtering surgeries such as trabeculectomy. Along with evidence of the good efficacy and safety profile of XEN45 in reducing IOP in glaucoma patients, the advantages and more importantly the long-term outcomes of combining XEN45 implantation with phacoemulsification compared with standalone XEN45 implantation remain a subject of ongoing investigation and debate. Limited evidence exists on the efficacy of XEN45 beyond 2 years of follow-up [1,2]. As clinicians strive to optimize outcomes for glaucoma patients, the importance of long-term follow-up after these procedures cannot be overstated.

In addition, registry-based studies, aiming at informing clinical decision-making and enhancing the quality of care provided to glaucoma patients undergoing surgical intervention contribute to shedding light on real-word practices and overcoming the limitations posed by stringent inclusion criteria and protocols of clinical trials. The Italian XEN-Glaucoma Registry (XEN-GTR) [3], set up for collecting data from nine centers in Italy, was created with the goal of gathering extensive prospective data on glaucoma patients having XEN45 implantation and its intra- and postoperative characteristics.

For these reasons, after a first evaluation of the efficacy and safety outcomes at 12 months of XEN45 alone or combined to phacoemulsification in glaucoma patients from the XEN-GTR [4], we extended the analysis to a follow-up period of 36 months.

This study aims at elucidating the long-term efficacy and safety in the real-world setting in glaucoma patients who have undergone XEN45 implantation alone or combined with phacoemulsification by reporting the 3-year outcomes of the XEN-GTR.

## 2. Materials and Methods

We conducted a registry-based investigation on glaucoma patients who underwent XEN45 implantation with or without phacoemulsification and were included in the Italian Glaucoma Registry, involving nine Italian centers. This study was conducted in accordance with the Declaration of Helsinki between 2018 and 2023. After being informed about the aim of the study, each patient provided written consent.

Patients with open angle glaucoma with medically uncontrolled IOP and/or intolerance to medical therapy were screened, and one eye per patient was consecutively included in the study. The following conditions had to be met in order for a patient to be eligible for enrollment in the XEN-GTR: (I) ≥18 years old; (II) diagnosis of open angle glaucoma, ref. [5] which included primary open angle glaucoma (POAG), pseudoexfoliation, and pigmentary glaucoma; and (III) willingness to follow the study protocol.

Patients were excluded if they had active ocular inflammation, conjunctival changes in the implant region, iris neovascularization, intolerance, or allergy to device components (glutaraldehyde or porcine derivatives). Secondary types of glaucoma (except for pseudoexfoliation and pigmentary glaucoma) were not included in the study. Prior failed glaucoma surgery was not considered and served as an exclusion criterion in addition to other systemic or ocular conditions except for those listed below.

Data from eyes enrolled in the XEN-GTR within three years after surgery were collected and examined. The study visits were performed at baseline (prior to the surgery), at day 1 and month 1, 3, 6, 12, 18, 24, 30, and 36 postoperatively. A Goldmann applanation tonometer was used to measure IOP at each study timepoint and the mean of two measurements or median of three measurements, if the difference between the two was >2 mmHg, was recorded. Visual acuity in decimals, the number of ocular hypotensive medications (OHMs), as well as complications and additional procedures were also recorded.

### 2.1. Surgical Technique

XEN45 implant is composed of glutaraldehyde-crosslinked porcine gelatin, with dimensions of 45 μm for the inner lumen, 150 μm for the external diameter, and 6 mm for length. A 0.1 mL injection of mitomycin C (MMC) at a dosage of 0.2 mg/mL was given under the conjunctiva prior to the start of the procedure. The XEN45 was inserted via the trabecular meshwork using a 27-gauge preloaded injector to create an artificial connection between the anterior chamber and the subconjunctival region. One experienced glaucoma surgeon per center, who had performed at least ten XEN45 implants, performed all the internal implants. When appropriate, the XEN45 implant procedure was combined with phacoemulsification. Because the XEN-GTR is a real-world based registry, study centers did not standardize the surgical technique; therefore, decisions on the position of the implant site and the type of surgery (combined or not in accordance with the patients’ eye characteristics) were left to each surgeon, as well as the postoperative management.

### 2.2. Statistical Analysis

The statistical analysis was conducted on the whole group of patients and separately for the XEN alone (eyes that underwent XEN45 implantation alone) and XEN+Phaco (eyes that underwent XEN45 implantation combined with phacoemulsification) groups.

The analysis was carried out following the recent recommendations of the European Glaucoma Society—A guide on surgical innovation for glaucoma [6]. The proportion of patients identified as surgical success after three years of follow-up served as the main outcome measure. The criteria for success included the following: IOP < 18 mmHg and IOP decrease ≥ 20% at 36 months, with (qualified success) or without OHMs (complete success). The total number of eyes that achieved qualified and complete successes indicated the overall success. Failure was defined as an IOP of >18 mmHg in two consecutive follow-up visits, an IOP reduction < 20% at month 36, further glaucoma surgery or surgical revision, or clinical hypotony (IOP < 6 mmHg and ≥2 lines of visual acuity loss after month 1). An IOP > 18 mmHg in the first follow-up month was not considered a failure criterion.

The open-source R (version 3.6.0) [7] with survival (version 3.2-7) [8] and survminer (version 0.4.9) [9] packages were used for statistical analysis.

Medians and interquartile ranges (IQRs) and numbers (%) were used to summarize continuous variables and categorical variables respectively. Two-sided Student *t* test or Mann–Whitney U test was used to compare continuous variables after checking for a normal distribution with the Shapiro–Wilk test. Categorical variables were tested with Chi-square or Fisher’s exact tests, as required. To test the IOP and OHMs changes during follow-up and differences between study groups, the repeated measures ANOVA (with the Greenhouse–Geisser correction) was used. To handle missing data, we conducted both per-protocol (PP) and a last observation carried forward (LOCF) analyses. The cumulative probability of success of surgery was tested with Kaplan–Meier survival curves, censoring patients who left the study before the end of follow-up. Conditional univariable and multivariable Cox hazard models were used to test the association between preoperative and intraoperative factors with surgery failure. Gender, age, right/left eye, glaucoma type, preoperative IOP, VF damage, and number of baseline OHMs were included as preoperative factors, whereas the surgery type, position of the surgeon, implant site, type of viscoelastic, and intraoperative complications as intraoperative factors. The martingale residuals method was used to test the validity of the proportional hazard’s prerequisites. A *p* value < 0.05 was considered statistically significant.

## 3. Results

Two hundred thirty-nine eyes of 239 patients from nine centers were analyzed, including 144 (60.2%) in the XEN alone group and 95 (39.8%) in the XEN+Phaco group. Of them, 87 eyes completed the 36 months of follow-up, including 53 and 34 in the XEN alone and XEN+Phaco groups, respectively. A total of 152 patients dropped out of follow-up before 36 months. Among them, five patients died before the end of follow-up, 43 patients ended follow-up because of the COVID-19 pandemic, eight patients underwent an additional glaucoma surgery, one patient developed endophthalmitis that caused the end of follow-up, and 95 patients ended their follow-up for unconfirmed/unknown reasons.

Table 1 summarizes the demographic and clinical characteristics of the patients enrolled.

The median (IQR) age of the patients was 72 (65–78) years, and the distribution of genders was balanced (44.3% females and 55.6% males). More than 90% of all the patients included were affected by POAG, without significant differences in the percentage of other types of glaucoma between the study groups (*p* = 0.28). Based on VF damage, 56 (23.4%), 63 (26.4%), and 103 (43.1%) eyes showed early, moderate, and severe glaucoma, respectively. The median (IQR) VF mean deviation (MD) was −11.0 (−18.0 to −5.2) dB.

### 3.1. Intraocular Pressure and Ocular Hypotensive Medications

**Figure 1:**
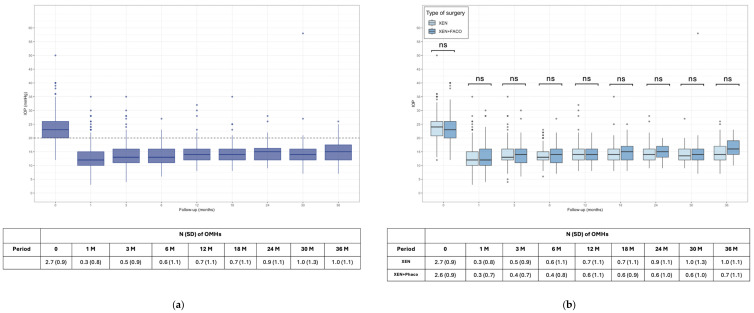
Boxplot showing the intraocular pressure (IOP) and number of ocular hypotensive medications (OHMs) at each timepoint of the follow-up for all patients (**a**) and for the XEN alone and XEN+Phaco groups (**b**). ns = not significant.

IOP and OHMs over the follow-up are depicted in Figure 1.

Over the follow-up period, mean preoperative IOP decreased from a median (IQR) of 23 (20–26) mmHg at baseline to 14.0 (12.0–16.0) mmHg at 12 months, 15 (12.0–16.2) mmHg at 24 months, and 15.0 (12.0–17.5) mmHg at 36 months (*p* < 0.01). Using LOCF, the analysis yielded same median values of median IOP except for at 24 and 36 months (14 mmHg with LOCF and 15 mmHg with PP) (Figure S1 and Table S1). In addition, the total range of IOP measurements at 36 months between LOCF (6 to 27 mmHg) and PP (7 to 26 mmHg) showed negligible differences.

Considering the two different study groups, no differences were found at any time point between IOP achieved with XEN and XEN+Phaco in the PP analysis. The LOCF analysis per group showed comparable results at each follow-up timepoint with a maximum difference of 1 mmHg, except for the XEN+Phaco group at 36 months, where the difference was 2 mmHg between the two analyses (14 mmHg with LOCF and 16 mmHg with PP) (Figure S1 and Table S1).

At month 36 the IOP reduction was −34.1 ± 20.1%, without statistically significant differences between the percentages achieved in the XEN alone (−36.9 ± 19.6%) and XEN+Phaco (−29.9 ± 20.4%) groups, *p* = 0.4.

**Figure 2:**
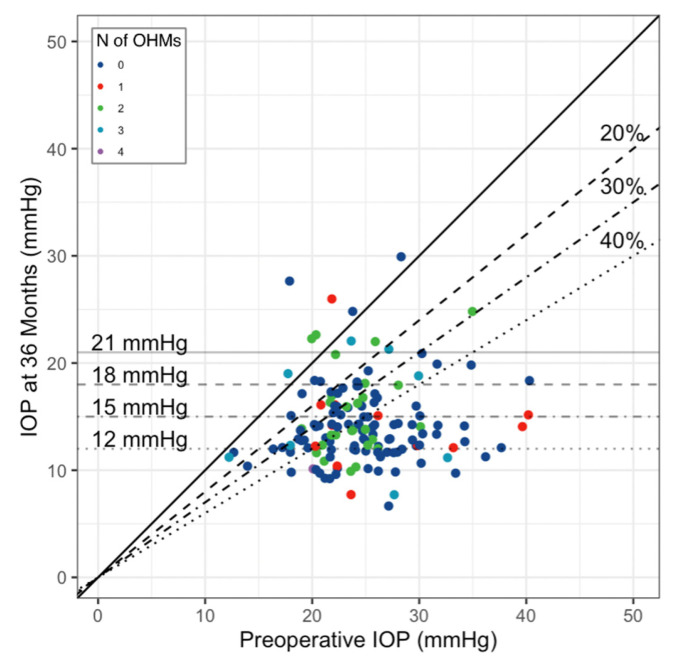
Scatterplot comparing IOP at baseline compared to the IOP at month 36 and the number of ocular hypotensive medications (LOCF analysis). Each point represents one eye. The IOP thresholds at month 36 are represented by the horizontal gray lines. The percentage levels of IOP reduction at month 36 in comparison to preoperative IOP are shown by the black lines. Eyes without IOP data are not displayed. IOP, intraocular pressure; OHM, ocular hypotensive medications. Differences between PP and LOCF analyses are reported in Figure S2.

The scatterplot in Figure 2 shows depicts the IOP reduction as a function of preoperative IOP and the number of OHMs at 36 months.

Month 36 saw 71 (81.6%), 52 (59.7%), and 36 (41.3%) eyes achieving reductions in preoperative IOP of at least 20%, 30%, and 40%, respectively. The percentages were slightly lower when using LOCF analysis: 177 (74.0%), 152 (63.6%), and 124 (51.8%), respectively. The IOP at 36 months was ≤21 mmHg in 79 (90.8%) eyes, ≤18 mmHg in 70 (80.4%) eyes, ≤15 mmHg in 51 (58.6%) eyes, and ≤12 mmHg in 24 (27.5%) eyes. The corresponding values for LOCF were 229 (96.8%), 213 (89.1%), 158 (66.1%), and 58 (24.2%) eyes.

Greater IOP reduction was achieved in patients with higher preoperative IOP ranging from −20.0% (−23.3–−14.1%) in eyes with IOP ≤ 15 mmHg to 62.5% (−65.0–−55.0%) in eyes with IOP ≥ 35 mmHg. However, the number of patients belonging to both ends of the ranges is small (Figure S3).

Complete data on ocular hypotensive medications (OHMs) were available only for 52 patients at 36 months of follow-up.

The mean number of OHMs was significantly lowered from 2.7 (0.95) at baseline to 0.94 (1.10) at 36 months (*p* < 0.001), without significant differences at any timepoint. With LOCF, the figures were comparable until 12 months of follow-up. Thereafter, LOCF showed slightly lower values of number of needed OHMs (0.64 vs. 0.94) (Figure S4 and Table S2).

The per-group LOCF analysis showed comparable values for the XEN+Phaco group. Subtle differences could be found in the XEN group where at 36 months the LOCF and PP values were 0.67 and 1.06 respectively.

A reduction in the number of eyes treated with 3 or 4 OHMs was observed over time, decreasing from 54.5% at baseline to 4.5% at 36 months. Notably, only 1 patient received 4 OHMs throughout the study (Figure S5). At 36 months, there were 117 (71.5%) OHM-free eyes (67.7% with the LOCF), including those treated with topical and systemic drugs. Additionally, among the patients with available data, the percentage of patients who received subsequent treatment with oral systemic carbonic anhydrase inhibitors decreased considerably from 84 (35.1%) to 0 (0.0%) (*p* < 0.001).

### 3.2. Effectiveness

**Figure 3:**
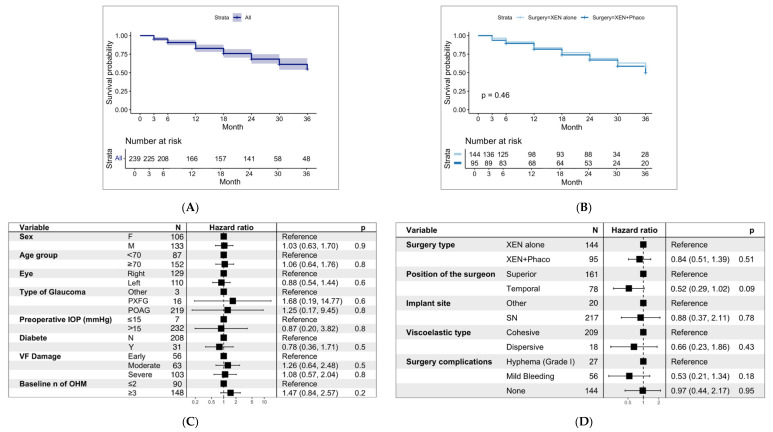
Kaplan–Meier survival curves illustrating the overall survival rates (**A**) and the survival rates for eyes treated with XEN alone and XEN+Phaco (**B**) and multivariate Cox proportional hazard ratios with 95% confidence intervals (CIs) for preoperative (**C**) and intraoperative (**D**) factors predictive of surgical failure. PXFG, pseudoexfoliation glaucoma; POAG, primary open angle glaucoma; VF, visual field; SN, superonasal.

According to the success definition, 161 (67.3%) eyes achieved overall success at the last available follow-up over 36 months. Of those, 106 (65.8%) eyes were classified as complete success and 55 (34.1%) as qualified success. Figure 3 depicts the Kaplan–Meier survival curves, illustrating the probability of success over time for the entire study sample (Figure 3A) and for the study groups (Figure 3B).

The XEN alone [69.4%, with 66 (66.0%) being complete success] and in the XEN+Phaco groups [64.2%, with 40 (68.8%) being complete success] showed comparable overall success rates (*p* = 0.07).

Among patients who adhered the success definition, 48 eyes reached 36 months of follow-up. At the end of the study, 44 were classified as overall success (91.6%) without statistically significant differences found between study groups. On the contrary, among patients who did not complete the follow-up, at their last available follow-up visit, 66 were classified as complete success, 25 as qualified success, and 61 as failure.

We further analyzed survival and success rates using various IOP thresholds: <12 mmHg, 15 mmHg, and 21 mmHg (Figure S6). At 36 months, success probabilities were similar between groups for the <12 mmHg threshold (XEN alone: 0.15, 95% CI: 0.09–0.25; XEN+Phaco: 0.07, 95% CI: 0.03–0.18, *p* = 0.56). However, for the <15 mmHg threshold, the XEN alone group had a significantly higher success rate (0.48, 95% CI: 0.38–0.61) compared to XEN+Phaco (0.27, 95% CI: 0.17–0.43, *p* = 0.041). When the threshold was set to <21 mmHg, both groups showed higher success probabilities (XEN alone: 0.66, 95% CI: 0.57–0.78; XEN+Phaco: 0.61, 95% CI: 0.49–0.76, *p* = 0.63) without significant differences.

A total of 78 (32.6%) eyes met the surgery failure criteria: one eye showed clinical hypotony, eight eyes needed an additional glaucoma procedure (one required cyclophotocoagulation, one required an additional XEN45 implant, four required trabeculectomy, one required a glaucoma drainage device, and one required a glaucoma micro shunt). Seven surgical revisions were performed, while 62 eyes did not achieve a sufficient IOP decrease.

The univariate and multivariate analyses (Figure 3) of factors associated with surgery failure showed that neither preoperative nor postoperative factors influence the success of the surgery.

### 3.3. Safety

Table 2 displays the intra- and postoperative complications that occurred during the follow-up, with the latter grouped by early (on or before month 1) and late (after month 1) events.

Each eye may have experienced single or multiple complications during follow-up.

Of the study population, nonserious AEs and SAEs were recorded in 57 (23.8%) and three (1.2%) patients, respectively. One hundred forty-six (61.1%) of the eyes experienced no intraoperative complications, whereas 64 (26.7%) eyes had mild bleeding, 29 (12.1%) had hyphema (Grade I), [10] and one (0.4%) eye had conjunctival tears. Regarding the postoperative complications, there were 91 eyes (38.1%) with at least one early complication and 56 eyes (23.4%) with at least one late complication. More than one postoperative complication occurred in 42 (17.5%) eyes, either during or after surgery.

The frequency of choroidal detachment was the highest [29 eyes (12.3%) and two eyes (0.8%) in the early and late follow-up period, respectively]. The majority of complications were self-limiting or treated without consequences on visual function. Among serious complications, two eyes (0.8%) with blebitis were registered at 6 months (effectively treated without clinical consequences), whereas one case of endophthalmitis was register at 18 months. Other cases of hypotony were mild and not clinically significant.

The subgroup analysis for rates of complications showed no difference in the early and late phases of follow-up between the XEN alone and the XEN+Phaco groups.

Additional procedures performed during the study follow-up are reported in Table 3.

During follow-up, 92 (38.5%) eyes needed at least one additional procedure. A total of 89 needlings were done on 55 (23.0%) eyes at least once throughout the follow-up period but no later than the first year. Thirty eyes required one needling, eighteen eyes required two, and two and five eyes required three and four needlings, respectively. In both XEN and XEN+Phaco groups, there were similar numbers of eyes that needed needlings [32 (13.2%) and 23 (9.6%), *p* = 0.5].

## 4. Discussion

This multicenter, prospective, and observational study centered on the XEN-GTR was conducted to evaluate the efficacy and safety outcomes over a 36-month period following the implantation of XEN45, either as a standalone procedure or in conjunction with phacoemulsification. Our findings demonstrate that both XEN45 implantation alone and in combination with phacoemulsification resulted in significant reductions in intraocular pressure (IOP) and a decreased requirement for ocular hypotensive medications (OHMs) in glaucoma patients over a long-term follow-up.

Over half of the eyes that concluded the study attained an IOP ≤ 15 mmHg at the conclusion of the follow-up period, with a mean reduction of 34.1%. Other reports exceeding 12 months of follow-up reported IOP reductions ranging from 27.8% and 29.3% at 24 months [1,11,12] to 37% at 36 months [2]. Our report is thus in line with what others have previously reported and further confirms the stability of IOP control over time and beyond the first year of surgery. Additionally, more than 50% of eyes did not require additional medical therapy to control IOP at the end of the follow-up with a mean number of OHMs of 0.9, slightly lower compared to that reported by Reitsamer et al. [1], whose patients started with a comparable number of medications (2.5 vs. 2.7 in the present study).

The overall success rate observed in our study was 68.1%. In our first report at 12 months of follow-up, the rate was 70.7%. This small difference suggests that the surgical effect of XEN45 combined or not with Phaco may last over an extended period in some patients. The success rate that we found was similar to that reported by Gillmann et al. (68.4%) for the same follow-up period of 36 months [2]. However, they used IOP ≤ 18 mmHg as a threshold instead of <18 mmHg used by our group. For this reason, comparisons of success rates should be made with caution.

Because of the variability in the definition of surgical success across various studies and the relative meaning of success in given patients, we also repeated the analysis with IOP < 12, <15 mmHg, and <21 mmHg, keeping the ≥20% IOP reduction in line with most updated recommendations [6]. With the more stringent < 12 mmHg threshold, the overall probability of success considerably decreases to 29.2% (31.1% and 25% for XEN alone and XEN+Phaco, respectively). For the <15 mmHg threshold, the overall success rate remained clinically significant at 57.3% (63.1% and 48.4% for XEN alone and XEN+Phaco, respectively). Increasing the threshold to ≤21 mmHg led to a higher success rate of 74.0% (75.6% and 71.5% for XEN alone and XEN+Phaco, respectively).

Regarding surgical failures, finding predictors may clinically translate into better tailored treatments for patients eligible for XEN or XEN+Phaco surgery. In our previous report at 12 months of follow-up [4], we found preoperative IOP < 15 mmHg and the temporal position of the surgeon to be significantly associated with surgery failure both in the univariate and multivariate analyses. On the contrary, no intraoperative or postoperative variables were found to be associated with surgery failure over 36 months of follow-up. The lack of identifiable factors associated with surgical failure at 3 years compared to 1 year may suggest a convergence of outcomes over time, reflecting and further supporting the long-term stability and efficacy of XEN implantation in selected patients. This may be further supported by the finding that the highest percentage of failures was registered during the first 12 months of follow-up (50%), followed by 37.1% between 12 and 24 months, and 12.8% up to 36 months, with a similar decreasing trend also previously described [2]. These observations may be attributed to several elements: (I) dynamics of bleb formation and stabilization; (II) suboptimal IOP control, especially in the first postoperative period; and (III) surgical complications. With respect to the first two points, several molecular processes underlie conjunctival fibrosis, from the release of proinflammatory cytokines to induce fibroblast proliferation and extracellular matrix remodeling [13,14]. Despite the widespread use of antifibrotic agents such us MMC and 5-fluorouracil in glaucoma bleb-forming surgeries, these processes may result in the formation of scarring and fibrotic blebs, with a consequent IOP increase, particularly in the first months of follow-up [15]. Lastly, regarding surgery complications, we registered a low number of postoperative complications with the highest frequency noted early during the follow-up (<month 1). Among the late complications, only one was registered after the first year of follow-up, in line with other reports with long follow-up [2,12]. Most complications showed similar frequencies except for corneal dellen and macular edema for which we observed somewhat higher rates. Nevertheless, these complications, as well as the serious ones, were successfully treated and did not show clinically significant consequences on patients’ visual function during the follow-up of the study.

A series of additional procedures have been also used during the follow-up to manage increased IOP. Approximatively 30% of the eyes needed at least one needling (all reported in the first 12 months of follow-up). These results are rather similar to the studies of Gabbay et al. [16], Reitsamer et al. [1], and Mansouri et al. [11] who reported a needling rate ranging between and 36.8% and 45%. However, the results differ appreciably from those reported by Tan et al. [17] and Rauchegger et al. [12] (51.3% and 60%, respectively).

The efficacy of XEN45 as a standalone procedure versus XEN+Phaco has been extensively examined over different follow-up lengths, yielding conflicting results regarding the superiority of one procedure compared to the other [4,11]. Consistent with previous investigations, we did not detect significant differences in success rates or IOP reduction between the two surgical procedures. Indeed, although the two surgeries were not formally compared statistically, both Gillman et al. [2] and Reitsamer et al. [1] concluded that no meaningful difference can be highlighted between the effectiveness of the XEN and XEN+Phaco procedures at 36 months of follow-up.

The principal strength of our study lies in its real-world, prospective design and the inclusion of a substantial number of eyes, enhancing the external generalizability of our results to the Caucasian population. However, some limitations need to be acknowledged. First and foremost, despite every attempt to retrieve missing data, a comparatively high percentage of patients were lost to follow-up. The highest number of patients (more than 40%) left the follow-up at 24 months, which corresponded approximately to 2020–2021. Although only 43 patients officially confirmed that they stopped their follow-up visits because of the COVID-19 pandemic, we speculate that it may have had a considerable effect on the loss to follow-up data. Furthermore, this study has inherent limitations of real-world studies, including a lack of randomization and variability in surgical techniques and decision-making. The heterogeneity in surgical procedures among participating centers underscores the need for cautious interpretation of effectiveness and overall success outcomes.

In our population, we did not include a group undergoing Phaco alone. Some studies reported often contradictory estimates of the effect of Phaco alone on IOP [18,19,20]. Therefore, although it is outside the scope of this study, no conclusions can be drawn about the magnitude of the effect of Phaco in addition to XEN45 implantation.

In conclusion, this study shows that over an extended follow-up, XEN45 implantation, either alone or combined with Phaco, is effective in stably reducing IOP and the need for additional OHMs in glaucoma patients, with comparable outcomes between standalone and combined procedures and a good safety profile.

## Figures and Tables

**Table 1:** Demographic and clinical characteristics.

	All Patients (*n* = 239)	XEN Alone (*n* = 144)	XEN+Phaco (*n* = 95)	*p* Value
**Age, median (IQR ^a^)**	72 (65–78)	73 (62–79)	72 (68–77)	0.57
**Gender** M F	133 (55.6%) 106 (44.3%)	77 (53.4%) 67 (46.5%)	56 (58.9%) 39 (41.0%)	0.46
**Ethnicity** Caucasian	239 (100%)	144 (100%)	95 (100%)	-
**Systemic conditions** Diabetes Hypertension Autoimmune diseases	31 (12.9%) 112 (77.7%) 14 (5.8%)	14 (9.7%) 66 (45.8%) 8 (5.5%)	17 (17.9%) 46 (48.4%) 6 (6.3%)	
**Type of glaucoma**				0.28
POAG ^b^ PXFG ^c^ Steroid induced Not reported	219 (91.6%) 16 (6.7%) 3 (1.2%) 1 (0.4%)	133 (92.3%) 8 (5.5%) 3 (2.0%) -	86 (90.5%) 8 (8.4%) - 1 (1.0%)	
**BCVA ^d^, median (IQR ^a^)**	0.5 (0.2–0.8)	0.8 (0.5–0.9)	0.5 (1.0–0.7)	<0.001
**IOP ^e^, median (IQR ^a^)**	23 (20–26)	24 (21–26)	23 (20–26)	0.37
**N of drugs, median (IQR ^a^)**	3 (2–3)	3 (2–3)	3 (2–3)	0.07
**Endothelial count, median (IQR ^a^)**	2120 (1870–2460)	2118 (1875–2376)	2224 (1870–256)	<0.001
**Lens state**	<0.001			
Phakic Pseudophakic	130 (54.4%) 109 (45.6%)	35 (24.3%) 109 (75.7%)	95 (100.0%) -	
**Previous surgery**	<0.001			
None Phaco SLT ^f^/ALT ^g^* Other procedures	124 (51.8%) 109 (45.6%) 5 (2.1%) 4 (1.6%)	33 (22.9%) 109 (75.7%) 4 (2.7%) 4 (2.7%)	94 (98.9%) - 1 (1.0%) -	
**Visual field damage**				
MD ^h^ (dB), median (IQR ^a^)	−11 (−18–−5.25)	−12 (−18–−6)	−10 (−16–−5)	0.24
Better than −6 dB −6 to −12 dB Worse than −12 dB Not reported	56 (23.4%) 63 (26.4%) 103 (43.1%) 17 (7.1%)	32 (22.2%) 32 (22.2%) 67 (46.6%) 13 (9.0%)	24 (25.3%) 31 (32.6%) 36 (37.9%) 4 (4.2%)	0.18
**C/D ^i^ ratio, median (IQR ^a^)**	0.7 (0.6–0.8)	0.7 (0.6–0.8)	0.7 (0.6–0.8)	0.48

^a^ IQR, interquartile range; **^b^** POAG, primary open angle glaucoma; ^c^ PXFG, pseudoexfoliation glaucoma; ^d^ BCVA, best corrected visual acuity; ^e^ IOP, intraocular pressure; ^f^ SLT, selective laser trabeculoplasty; ^g^ ALT, argon laser trabeculoplasty; ^h^ MD, mean deviation; ^i^ C/D, cup to disc. * Performed as standalone procedure or in patients with history of previous procedures.

**Table 2:** Early and late complications.

	Early (Before Month 1)				Late (After Month 1)			
	All Patients (*n* = 239)	XEN Alone (*n* = 144)	XEN+Phaco (*n* = 95)	*p* Value	All Patients (*n* = 239)	XEN Alone (*n* = 144)	XEN+Phaco (*n* = 95)	*p* Value
Corneal edema	5 (2.1%)	2 (1.4%)	3 (3.1%)	0.24	9 (3.7%)	5 (3.5%)	4 (4.2%)	0.07
Corneal dellen	0 (0.0%)	0 (0.0%)	0 (0.0%)		9 (3.7%)	4 (2.7%)	5 (5.2%)	
Shallow AC ^a^	17 (7.1%)	10 (6.9%)	7 (7.4%)		0 (0.0%)	0 (0.0%)	0 (0.0%)	
Hyphema	12 (5.0%)	5 (3.5%)	7 (7.4%)		0 (0.0%)	0 (0.0%)	0 (0.0%)	
Iritis	0 (0.0%)	0 (0.0%)	0 (0.0%)		7 (2.9%)	4 (2.7%)	3 (3.1%)	
AC ^a^ flare	0 (0.0%)	0 (0.0%)	0 (0.0%)		3 (1.6%)	2 (1.4%)	1 (1.0%)	
XEN45 blocked lumen	16 (6.7%)	7 (4.8%)	9 (9.5%)		12 (6.1%)	5 (3.5%)	7 (7.4%)	
Dislocated device	0 (0.0%)	0 (0.0%)	0 (0.0%)		2 (0.8%)	1 (0.7%)	1 (1.0%)	
Conjunctival tear	0 (0.0%)	0 (0.0%)	0 (0.0%)		2 (0.8%)	1 (0.7%)	1 (1.0%)	
Leak/dehiscence	5 (2.1%)	3 (2.1%)	2 (2.1%)		0 (0.0%)	0 (0.0%)	0 (0.0%)	
Conjunctival erosion	0 (0.0%)	0 (0.0%)	0 (0.0%)		3 (1.2%)	2 (1.4%)	1 (1.0%)	
Choroidal detachment	29 (12.3%)	14 (9.7%)	15 (15.7%)		2 (0.8%)	2 (1.4%)	0 (0.0%)	
Hypotony maculopathy	11 (4.6%)	6 (4.2%)	5 (5.2%)		5 (2.1%)	3 (2.1%)	2 (2.1%)	
Macular edema	0 (0.0%)	0 (0.0%)	0 (0.0%)		9 (3.7%)	4 (2.7%)	5 (5.2%)	
Diplopia	0 (0.0%)	0 (0.0%)	0 (0.0%)		2 (0.8%)	1 (0.7%)	1 (1.0%)	
Ptosis	0 (0.0%)	0 (0.0%)	0 (0.0%)		3 (1.2%)	1 (0.7%)	2 (2.1%)	
Serious complications								
Blebitis	0 (0.0%)	0 (0.0%)	0 (0.0%)	-	2 (0.8%)	1 (0.7%)	1 (1.0%)	-
Endophthalmitis	0 (0.0%)	0 (0.0%)	0 (0.0%)		1 (0.4%)	1 (0.7%)	0 (0.0%)	

^a^ AC, anterior chamber.

**Table 3:** Additional procedures.

Procedure *, *n* (%)	Total, *n* (%) (*n* = 239)
Needling	
Alone +MMC ^a^ +5FU ^b^ +Corticosteroid +Lidocaine	27 (11.3%) 18 (7.5%) 14 (5.8%) 7 (2.9%) 2 (0.8%)
Manual subconjunctival mobilization	28 (11.7%)
Digital massage	19 (7.9%)
XEN45 removal and reimplant	4 (1.6%)
Bleb drainage + contact lens	1 (0.4%)
Other procedures	
Laser for luminal obstruction Conjunctival Suture IOL repositioning + reimplantation Anterior chamber refill with viscoelastic Trabeculectomy Glaucoma drainage device Glaucoma micro shunt Cyclophotocoagulation Second XEN45 implant	1 (0.4%) 1 (0.4%) 1 (0.4%) 1 (0.4%) 4 (1.6%) 1 (0.4%) 1 (0.4%) 1 (0.4%) 1 (0.4%)

^a^ MMC, mitomycin-C; ^b^ 5-fluorouracil. * During follow-up, eyes may have had treatment with a single procedure, multiple treatments, or a combination of procedures.

## Data Availability

Upon reasonable request, the corresponding author will provide the data gathered and/or analyzed during the current work.

## Supplementary Materials

The following supporting information can be downloaded at: https://www.mdpi.com/article/10.3390/jcm13237370/s1, Figure S1: Intraocular pressure (IOP) reduction over follow-up time with per-protocol (PP) and last observation carried forward (LOCF) analyses in all patients (a) and the XEN and XEN+Phaco groups. Figure S2: Scatterplot of the IOP at the preoperative visit compared to the IOP at month 36 based on the preoperative IOP and the number of ocular hypotensive medications with PP (a) and LOCF analyses (b). Figure S3: Intraocular pressure (IOP) reduction (%) according to baseline IOP ranges. Figure S4: Reduction in the need for ocular hypotensive medications (OHMs) over follow-up time with per-protocol (PP) and last observation carried forward (LOCF) analyses in all patients. Figure S5: Percentage of patients treated without or with 1, 2, 3, and 4 ocular hypotensive medications at baseline compared to 36 months of follow-up. Figure S6: Survival curves with different thresholds of IOP for definition of surgical failure: 12 mmHg (a), 15 mmHg (b), and 21 mmHg (c). Table S1: Values of intraocular pressure (IOP) over follow-up time with per-protocol (PP) and last observation carried forward (LOCF) analyses in all patients, XEN and XEN+Phaco groups. Table S2: Reduction in the need for ocular hypotensive medications (OHMs) over follow-up time with per-protocol (PP) and last observation carried forward (LOCF) analyses in all patients.

## References

[1] Reitsamer H., Vera V., Ruben S., Au L., Vila-Arteaga J., Teus M., Lenzhofer M., Shirlaw A., Bai Z., Balaram M. (2022). Three-Year Effectiveness and Safety of the XEN Gel Stent as a Solo Procedure or in Combination with Phacoemulsification in Open-Angle Glaucoma: A Multicentre Study. Acta Ophthalmol..

[2] Gillmann K., Bravetti G.E., Rao H.L., Mermoud A., Mansouri K. (2021). Combined and Stand-Alone XEN 45 Gel Stent Implantation: 3-Year Outcomes and Success Predictors. Acta Ophthalmol..

[3] Posarelli C., Figus M., Roberti G., Giammaria S., Ghirelli G., Quercioli P., Micelli Ferrari T., Pace V., Mastropasqua L., Agnifili L. (2022). Italian Candidates for the XEN Implant: An Overview from the Glaucoma Treatment Registry (XEN-GTR). J. Clin. Med..

[4] Oddone F., Roberti G., Giammaria S., Posarelli C., Ghirelli G., Mastropasqua L., Agnifili L., Micelli Ferrari T., Pace V., Nucci P. (2024). Effectiveness and Safety of XEN45 Implant over 12 Months of Follow-up: Data from the XEN-Glaucoma Treatment Registry. Eye.

[5] (2021). European Glaucoma Society Terminology and Guidelines for Glaucoma, 5th Edition. Br. J. Ophthalmol..

[6] Abegao Pinto L., Sunaric Mégevand G., Stalmans I., Azuara-Blanco A., Bron A., Garcia Feijoo J., Garway Heath T., Grehn F., King A., Kirwan J. (2023). European Glaucoma Society—A Guide on Surgical Innovation for Glaucoma. Br. J. Ophthalmol..

[7] R Core Team (2019). R: A Language and Environment for Statistical Computing.

[8] Therneau T.M., Grambsch P.M. (2000). Modeling Survival Data: Extending the Cox Model.

[9] Therneau T.M. (2020). A Package for Survival Analysis in R. https://CRAN.R-project.org/package=survival.

[10] Edwards W.C., Layden W.E. (1973). Traumatic Hyphema. A Report of 184 Consecutive Cases. Am. J. Ophthalmol..

[11] Mansouri K., Bravetti G.E., Gillmann K., Rao H.L., Ch’ng T.W., Mermoud A. (2019). Two-Year Outcomes of XEN Gel Stent Surgery in Patients with Open-Angle Glaucoma. Ophthalmol. Glaucoma.

[12] Rauchegger T., Angermann R., Willeit P., Schmid E., Teuchner B. (2021). Two-Year Outcomes of Minimally Invasive XEN Gel Stent Implantation in Primary Open-Angle and Pseudoexfoliation Glaucoma. Acta Ophthalmol..

[13] Lockwood A., Brocchini S., Khaw P.T. (2013). New Developments in the Pharmacological Modulation of Wound Healing after Glaucoma Filtration Surgery. Curr. Opin. Pharmacol..

[14] Skuta G.L., Parrish R.K. (1987). Wound Healing in Glaucoma Filtering Surgery. Surv. Ophthalmol..

[15] Jung K.I., Park H., Jung Y., Park C.K. (2015). Serial Changes in the Bleb Wall after Glaucoma Drainage Implant Surgery: Characteristics during the Hypertensive Phase. Acta Ophthalmol..

[16] Gabbay I.E., Allen F., Morley C., Pearsall T., Bowes O.M., Ruben S. (2020). Efficacy and Safety Data for the XEN45 Implant at 2 Years: A Retrospective Analysis. Br. J. Ophthalmol..

[17] Tan S.Z., Walkden A., Au L. (2018). One-Year Result of XEN45 Implant for Glaucoma: Efficacy, Safety, and Postoperative Management. Eye.

[18] Chen P.P., Lin S.C., Junk A.K., Radhakrishnan S., Singh K., Chen T.C. (2015). The Effect of Phacoemulsification on Intraocular Pressure in Glaucoma Patients: A Report by the American Academy of Ophthalmology. Ophthalmology.

[19] Baek S.U., Kwon S., Park I.W., Suh W. (2019). Effect of Phacoemulsification on Intraocular Pressure in Healthy Subjects and Glaucoma Patients. J. Korean Med. Sci..

[20] Ahmed I.I.K., De Francesco T., Rhee D., McCabe C., Flowers B., Gazzard G., Samuelson T.W., Singh K. (2022). Long-Term Outcomes from the HORIZON Randomized Trial for a Schlemm’s Canal Microstent in Combination Cataract and Glaucoma Surgery. Ophthalmology.

