# Supplementary material for: Italian XEN-Glaucoma Treatment Registry (XEN-GTR): Effectiveness and Safety at 36 Months of XEN45 Implant
Source: J Clin Med. 2024 Dec 3;13(23):7370. doi: 10.3390/jcm13237370 (PMC11641929; doi:10.3390/jcm13237370)
Supplement: Supplementary file 1 [file jcm-13-07370-s001.zip › Supplementary Files/Supplementary Tables.pdf]

Table S1. Values of Intraocular pressure (IOP) over follow-up time with per protocol (PP) and last observation carried forward (LOCF) analysis in all patients, XEN and XEN+Phaco groups.

	**All Patients**		**XEN**		**XEN+Phaco**	
**Period**	**LOCF** IOP Median (IQR)	**PP** IOP Median (IQR)	**LOCF** IOP Median (IQR)	**PP** IOP Median (IQR)	**LOCF** IOP Median (IQR)	**PP** IOP Median (IQR)
0 (Baseline)	23 (20 – 26)	23 (20 – 26)	24 (21.3 – 26.6)	24 (21.3 – 26.6)	23 (20 – 26)	23 (20 – 26)
1	12 (9.5 – 14.5)	12 (9.5 – 14.5)	12 (9.5 – 14.5)	12 (9.5 – 14.5)	12 (9 – 15)	12 (9 – 15)
3	13 (10.5 – 15.5)	13 (10.5 – 15.5)	13 (11 – 15)	13 (11 – 15)	14 (11.5 – 16.5)	14 (11.5 – 16.5)
6	13 (10.5 – 15.5)	13 (10.5 – 15.5)	13 (11.5 – 14.5)	13 (11.5 – 14.5)	14 (11.5 – 16.5)	14 (11.5 – 16.5)
12	14 (12 – 16)	14 (12 – 16)	13 (11 – 15)	14 (12 – 16)	14 (11.5 – 16.5)	14 (12 – 16)
18	14 (12 – 16)	14 (12 – 16)	14 (12 – 16)	14 (12 – 16)	14 (12 – 16)	15 (12.5 – 17.5)
24	14 (11.8 – 16.1)	15 (12.8 – 17.1)	14 (12 – 16)	14 (12 – 16)	14 (11.7 – 16.2)	15 (13 – 17)
30	14 (11.5 – 16.5)	14 (12 – 16)	14 (12 – 16)	13.5 (11.5 – 15.5)	14 (11.5 – 16.5)	14 (12 – 16)
36	14 (11.5 – 16.5)	15 (12.2 – 17.7)	14 (12 – 16)	14 (11.5 – 16.5)	14 (11.5 – 16.5)	16 (13.5 – 18.5)

Table S2: Ocular hypotensive medications (OHM) need reduction over follow-up time with per protocol (PP) and last observation carried forward (LOCF) analysis in all patients.

	**All Patients**		**XEN**		**XEN+Phaco**	
**Period**	**LOCF** N of OHMs Mean (SD)	**PP** N of OHMs Mean (SD)	**LOCF** N of OHMs Mean (SD)	**PP** N of OHMs Mean (SD)	**LOCF** N of OHMs Mean (SD)	**PP** N of OHMs Mean (SD)
0 (Baseline)	2.77 (0.92)	2.77 (0.92)	2.75 (0.92)	2.75 (0.92)	2.81 (0.91)	2.81 (0.91)
1	0.32 (0.76)	0.32 (0.76)	0.35 (0.83)	0.35 (0.82)	0.27 (0.62)	0.27 (0.62)
3	0.44 (0.85)	0.44 (0.85)	0.50 (0.92)	0.50 (0.93)	0.36 (0.73)	0.36 (0.73)
6	0.55 (0.99)	0.53 (0.97)	0.63 (1.10)	0.61 (1.12)	0.43 (0.80)	0.41 (0.75)
12	0.62 (1.01)	0.60 (1.01)	0.70 (1.11)	0.69 (1.11)	0.50 (0.84)	0.47 (0.80)
18	0.58 (1.00)	0.70 (1.11)	0.57 (1.00)	0.74 (1.14)	0.58 (1.02)	0.63 (1.07)
24	0.60 (1.00)	0.83 (1.16)	0.62 (1.05)	0.92 (1.24)	0.57 (0.93)	0.65 (0.98)
30	0.61 (1.01)	0.90 (1.21)	0.62 (1.05)	1.05 (1.31)	0.58 (0.96)	0.63 (1.02)
36	0.64 (1.00)	0.94 (1.10)	0.67 (1.03)	1.06 (1.11)	0.61 (0.98)	0.77 (1.10)
