# Supplementary figures and images for: Italian XEN-Glaucoma Treatment Registry (XEN-GTR): Effectiveness and Safety at 36 Months of XEN45 Implant
Source: J Clin Med. 2024 Dec 3;13(23):7370. doi: 10.3390/jcm13237370 (PMC11641929; doi:10.3390/jcm13237370)

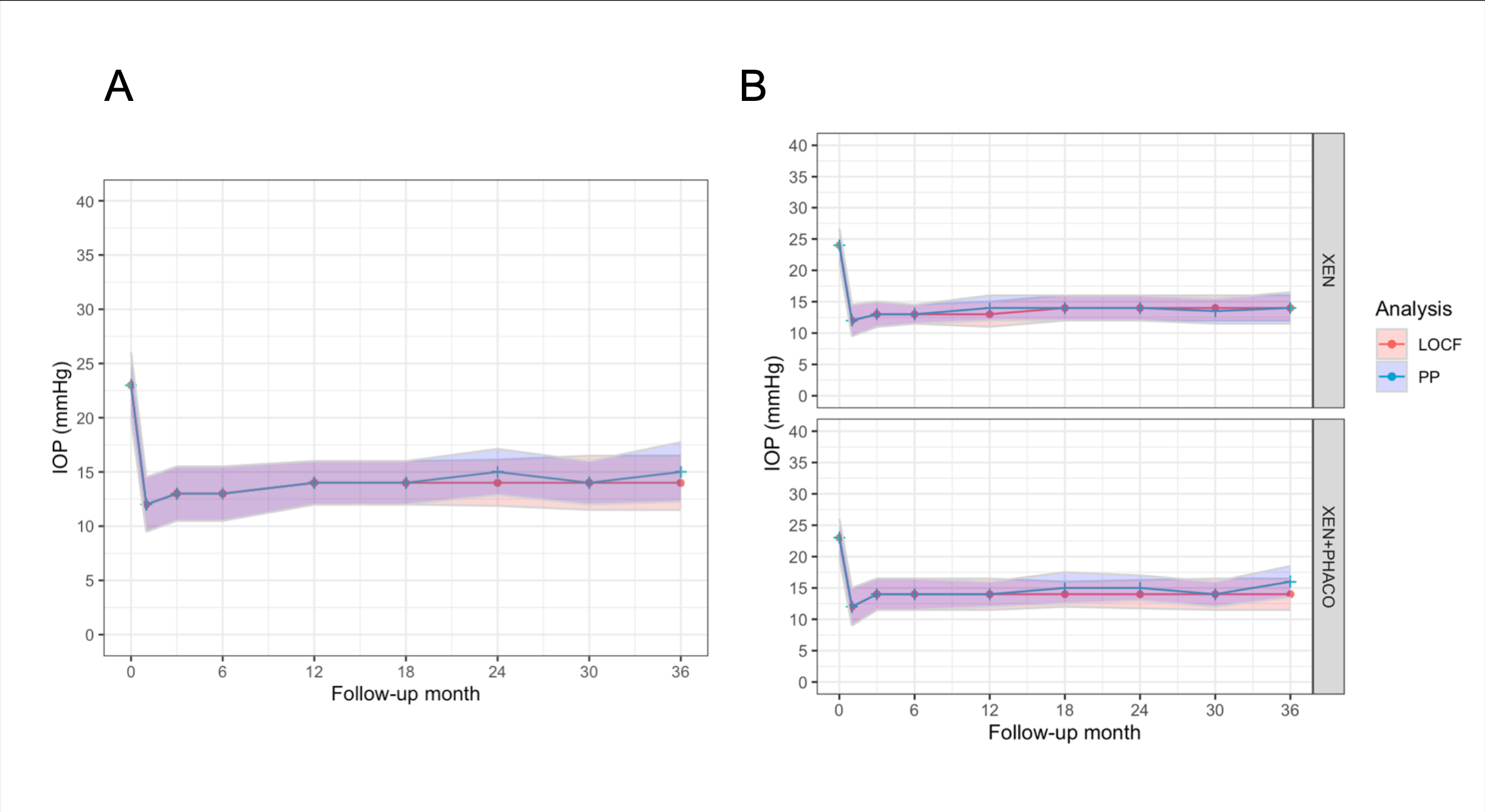

Supplement: Supplementary file 1 [file jcm-13-07370-s001.zip › Supplementary Files/Figure S1.tiff]

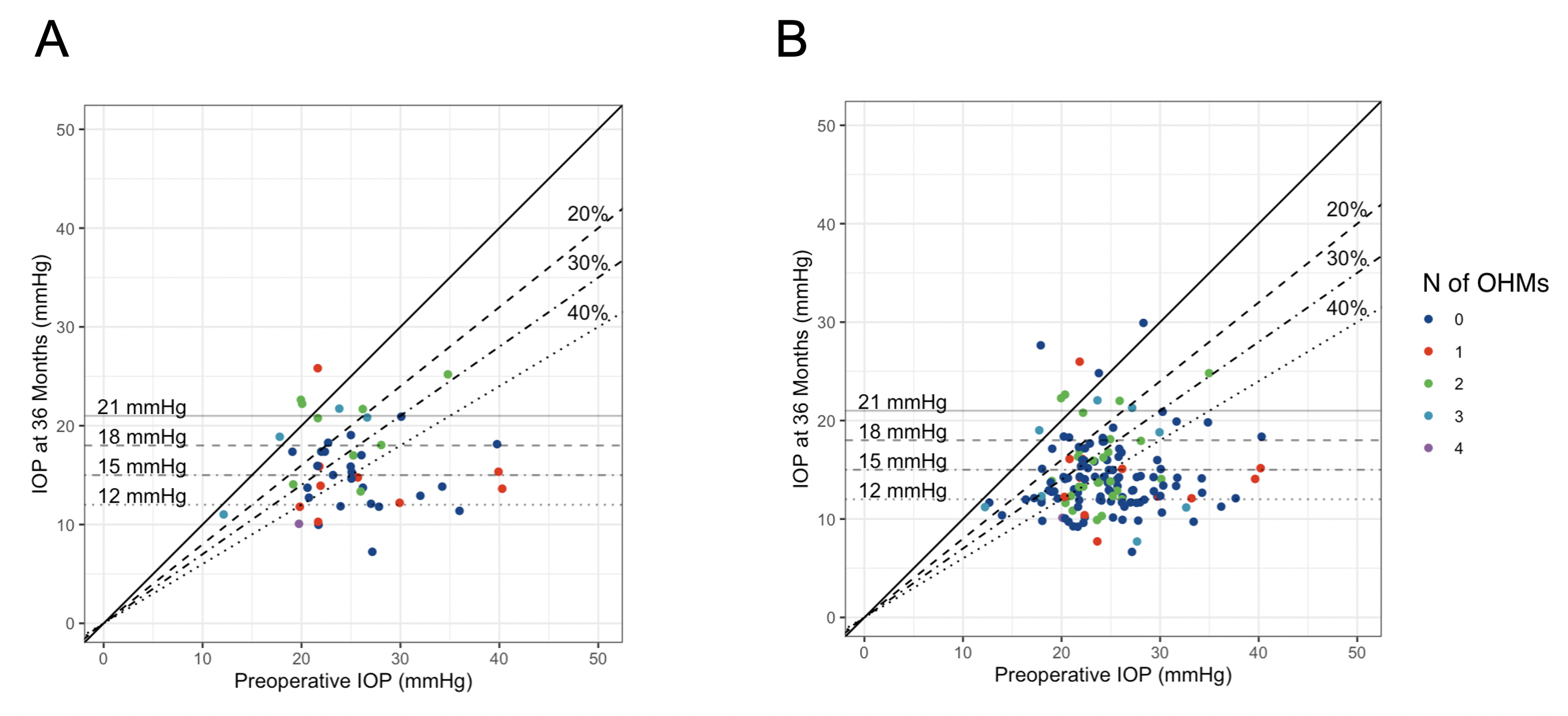

Supplement: Supplementary file 1 [file jcm-13-07370-s001.zip › Supplementary Files/Figure S2.tiff]

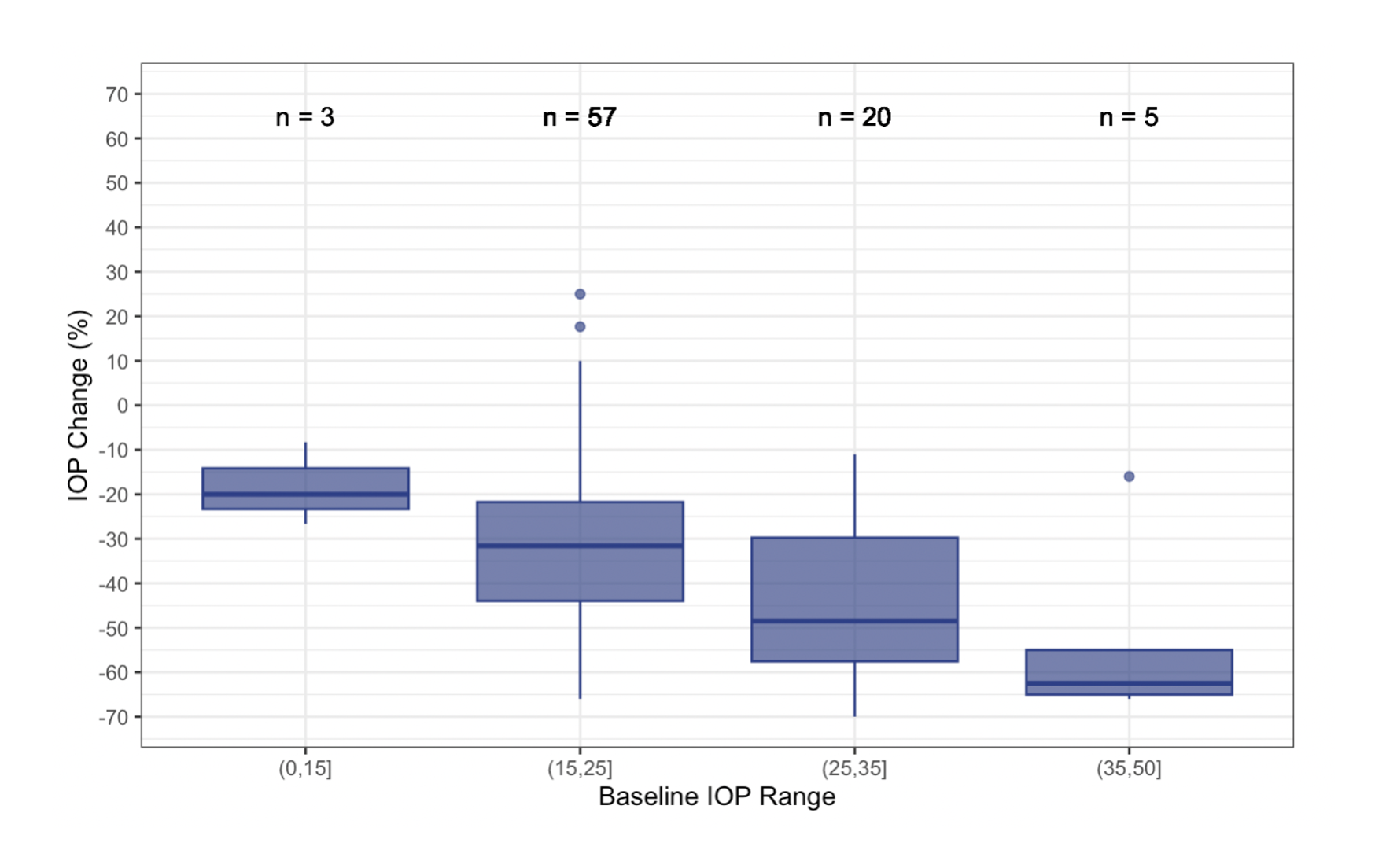

Supplement: Supplementary file 1 [file jcm-13-07370-s001.zip › Supplementary Files/Figure S3.png]

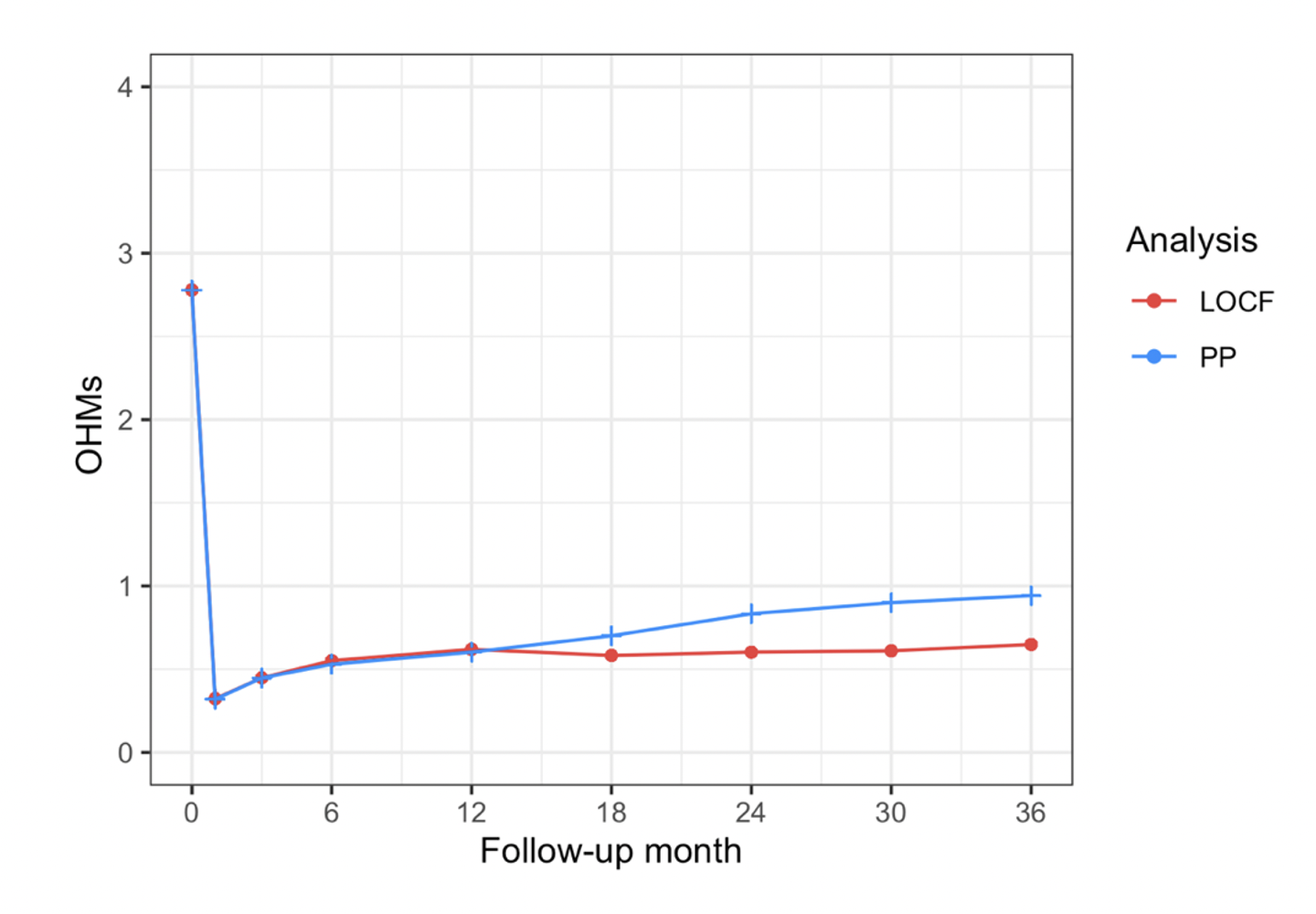

Supplement: Supplementary file 1 [file jcm-13-07370-s001.zip › Supplementary Files/Figure S4.png]

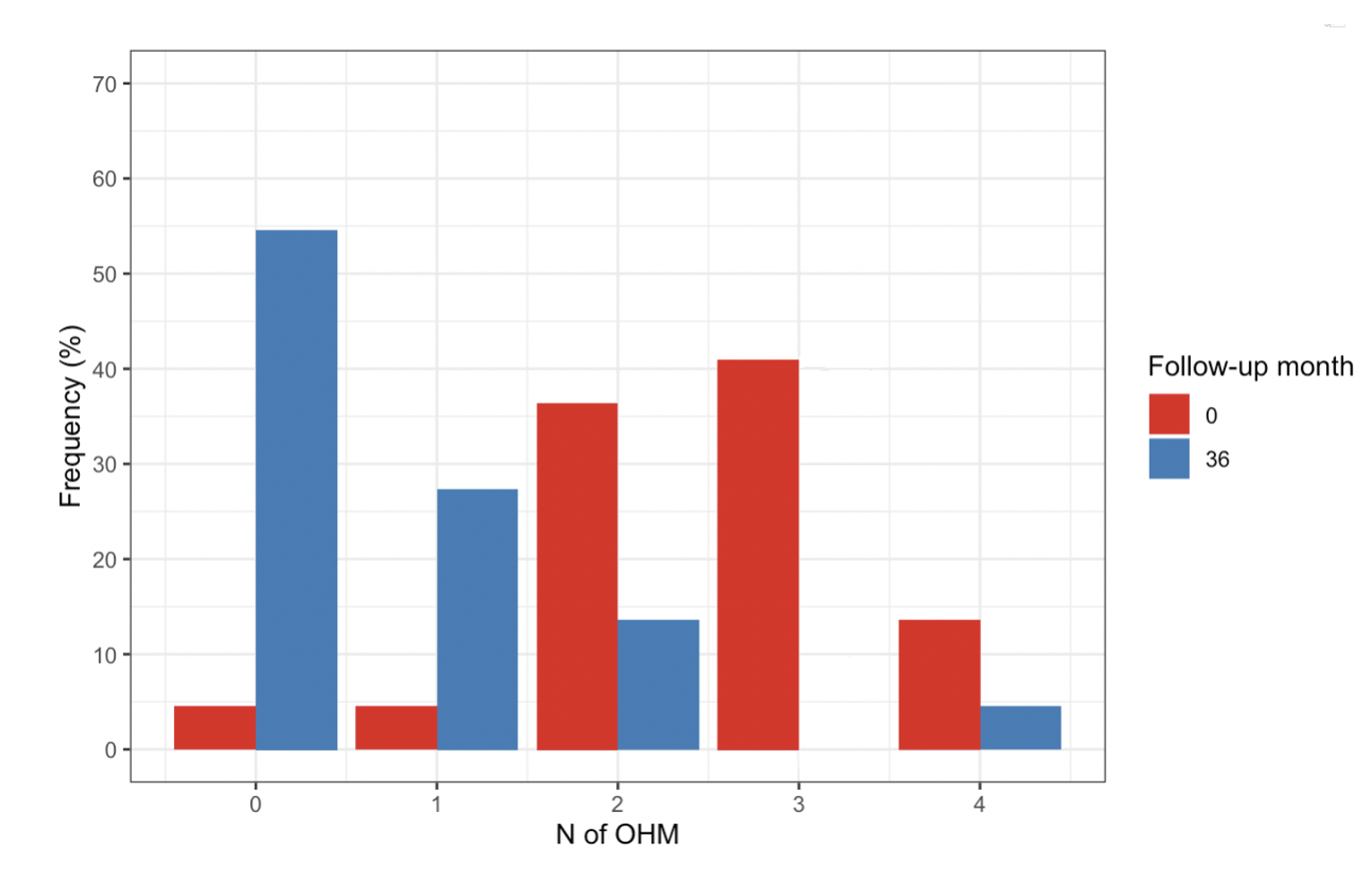

Supplement: Supplementary file 1 [file jcm-13-07370-s001.zip › Supplementary Files/Figure S5.png]

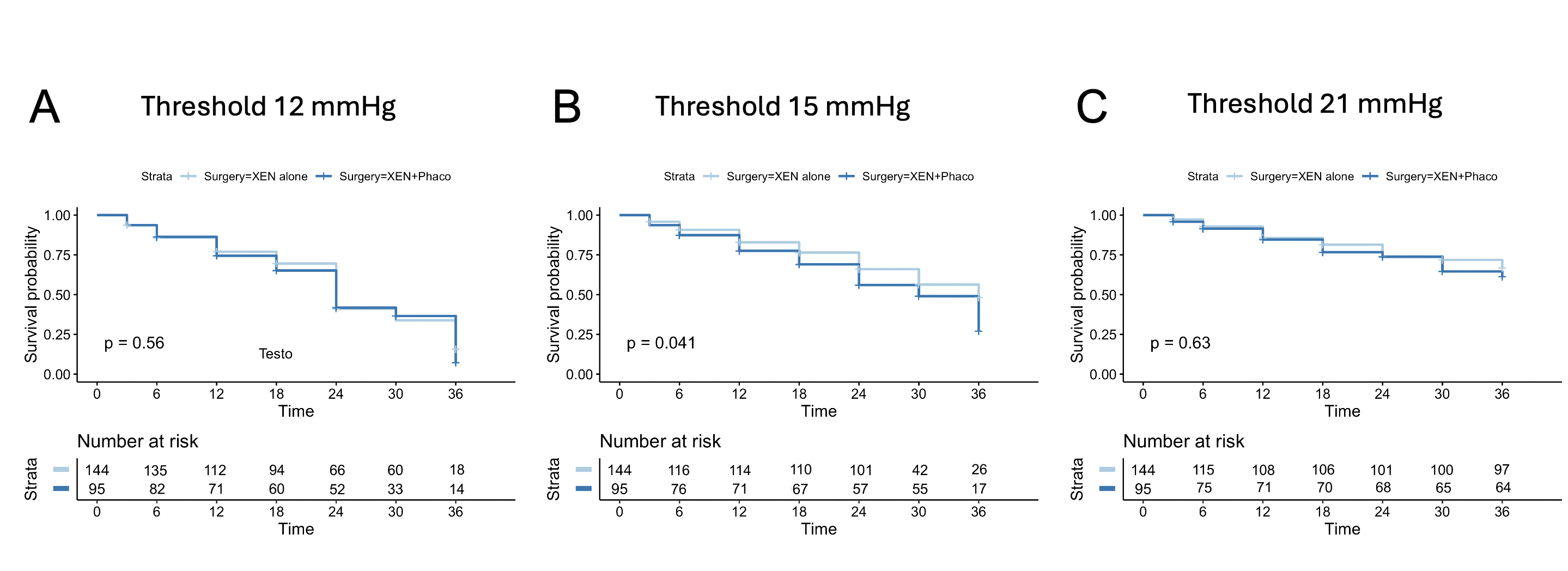

Supplement: Supplementary file 1 [file jcm-13-07370-s001.zip › Supplementary Files/Figure S6.png]
